# Effects of Long-Chain Polyunsaturated Fatty Acids in Combination with Lutein and Zeaxanthin on Episodic Memory in Healthy Older Adults

**DOI:** 10.3390/nu15132825

**Published:** 2023-06-21

**Authors:** Toshiaki Sueyasu, Keisuke Yasumoto, Hisanori Tokuda, Yoshihisa Kaneda, Hidenori Obata, Tomohiro Rogi, Takayuki Izumo, Sumio Kondo, Jiro Saito, Takashi Tsukiura, Masaaki Nakai

**Affiliations:** 1 Institute for Science of Life, Suntory Wellness Limited, 8-1-1 Seikadai, Seika-cho, Soraku-gun, Kyoto 619-0284, Japan; keisuke_yasumoto@suntory.co.jp (K.Y.); hisanori_tokuda@suntory.co.jp (H.T.); yoshihisa_kaneda@suntory.co.jp (Y.K.); hidenori_obata@suntory.co.jp (H.O.); tomohiro_rogi@suntory.co.jp (T.R.); takayuki_izumo@suntory.co.jp (T.I.); masaaki_nakai@suntory.co.jp (M.N.); 2 Fukushima Healthcare Center, Medical Corporation Kenshokai, 2-12-16, Tamagawa, Fukushima-ku, Osaka 553-0044, Japan; s.kondo@drc-web.co.jp; 3 Medical Station Clinic, 3-12-8, Takaban, Meguro-ku, Tokyo 152-0004, Japan; j.saito@med-station.jp; 4 Department of Cognitive, Behavioral and Health Sciences, Graduate School of Human and Environmental Studies, Kyoto University, Yoshida Nihonmatsu-cho, Sakyo-ku, Kyoto 606-8501, Japan; tsukiura.takashi.6c@kyoto-u.ac.jp

**Keywords:** arachidonic acid, docosahexaenoic acid, eicosapentaenoic acid, long-chain polyunsaturated fatty acids, lutein, zeaxanthin, episodic memory

## Abstract

Arachidonic acid (ARA), docosahexaenoic acid (DHA), and eicosapentaenoic acid (EPA), which are long-chain polyunsaturated fatty acids (LCPUFAs), as well as lutein (L) and zeaxanthin (Z), can potentially improve brain function. However, the effect of a combination of these components (LCPUFAs + LZ) on memory function in healthy older individuals remains unclear. This study aimed to determine if LCPUFAs + LZ-supplemented food could improve memory function. Exploratory and confirmatory trials (Trials 1 and 2, respectively) were conducted in healthy older Japanese individuals with memory complaints. We conducted randomized, double-blind, placebo-controlled, parallel-group trials. Participants were randomly allocated to two groups: placebo or LCPUFAs + LZ. LCPUFAs + LZ participants were provided with supplements containing ARA, DHA, EPA, L, and Z for 24 weeks in Trial 1 and 12 weeks in Trial 2. Memory functions were evaluated using Cognitrax before and after each trial. Combined analyses were performed for subgroups of participants with cognitive decline in Trials 1 and 2. The results showed that supplementation with LCPUFAs + LZ did not significantly affect memory function in healthy, non-demented, older individuals with memory complaints whereas it improved memory function in healthy, non-demented, older individuals with cognitive decline.

## 1. Introduction

Dementia is a serious social problem globally, and thus, preventing cognitive decline is important. A well-balanced diet is crucial for maintaining cognitive function [1]; many studies have been conducted on the effects of food components on memory function, which is a major cognitive function. Arachidonic acid (ARA), docosahexaenoic acid (DHA), and eicosapentaenoic acid (EPA), which are long-chain polyunsaturated fatty acids (LCPUFAs), are contained in fish, meat, and eggs. Carotenoids, including lutein (L) and zeaxanthin (Z), are contained mainly in green vegetables.

ARA and DHA are abundant in the brain and are major components of phospholipids. The amount of these fatty acids in the brain decreases with aging [2,3,4] but can be restored by supplementation [4]. Supplementation with ARA, DHA, and EPA may have a beneficial effect on cognitive function, as high doses of DHA and EPA reportedly improve memory function in older individuals [5,6]. However, to the best of our knowledge, there are no reports on the effects of ARA on memory function in healthy older individuals.

Among more than 1000 carotenoids found in nature [7], 40–50 are present in the human diet [8,9] and 6 are mainly found in human blood [10]. Among them, lutein and zeaxanthin (LZ) are concentrated in the center of the retina, which is called the macula, and have been reported to protect the optic nerve from oxidation and inflammation owing to their antioxidant and anti-inflammatory effects [11,12,13,14]. LZ are antioxidant components in the brain [15] and have been reported to have a protective effect on nerve cells [16,17,18,19], thereby affecting cognitive function. Indeed, it has been reported that LZ intake improves memory function in young people [20]. However, the efficacy of lutein and zeaxanthin on memory function is unclear due to inconsistent results from previous intervention studies [21,22].

In addition, the effects of DHA and EPA supplementation on memory function have had no effect in previous studies on healthy older individuals without cognitive decline [23,24]. However, positive effects have been observed in participants with cognitive decline, such as age-related cognitive decline [5] and mild cognitive impairment (MCI) [6]. These previous findings suggest that supplementation with food components is more likely to improve the memory function of participants with cognitive decline than of those with normal cognitive function.

Based on the facts that ARA, DHA, EPA, L, and Z (LCPUFAs + LZ) are present in the brain and several reports suggest memory function improvement with some components, a combination of these components may improve memory function in healthy older individuals. However, no previous studies have confirmed their effects. Therefore, in the present study, the following two hypotheses were formulated. Hypothesis 1: Food supplements containing LCPUFAs + LZ improve memory function in healthy older individuals with memory complaints. Hypothesis 2: Memory function is more likely to improve by LCPUFAs + LZ in participants with cognitive decline than in participants with normal cognitive function. For the exploratory examination of Hypothesis 1, an exploratory trial (Trial 1) was conducted in healthy older individuals with memory complaints. A confirmatory trial (Trial 2) was conducted after Trial 1 was deemed to be meaningful. To examine Hypothesis 2, a subgroup analysis of participants with cognitive decline in Trials 1 and 2 was performed. Combined analyses for subgroups of participants with cognitive decline in Trials 1 and 2 were also performed to comprehensively interpret the results of the two individual subgroup analyses with a larger sample size than that of the individual trials.

## 2. Materials and Methods

### 2.1. Study Design of Trial 1

A randomized, double-blind, placebo-controlled, parallel-group trial was designed to perform an exploratory evaluation of the effect of supplementation with LCPUFAs + LZ on memory function in healthy older Japanese individuals with memory complaints without dementia. This trial was conducted between April 2019 and February 2020 at a medical institution in Meguro-ku, Tokyo, Japan. A total of 776 participants were recruited from Tokyo and neighboring regions, of whom 120 were enrolled and randomly allocated to three groups: (1) a placebo group, receiving a placebo as a food supplement; (2) an LCPUFAs + X group, receiving a food supplement consisting of LCPUFAs (containing 120 mg ARA, 300 mg DHA, and 100 mg EPA per day) combined with compound X (whose contents are not shown because this compound is not the subject of this study); (3) an LCPUFAs + LZ group, receiving a food supplement consisting of LCPUFAs (containing 120 mg ARA, 300 mg DHA, and 100 mg EPA per day) combined with LZ (containing 10 mg lutein and 2 mg zeaxanthin per day). The intervention period was set at 24 weeks, based on previous studies that evaluated memory function using food components and considering that the potential for efficacy could be determined after less than 24 weeks of consumption [5,25,26]. Fasting hematology, blood biochemistry, urinalysis, infectious disease test results, and physical examination values were used to select healthy participants. Both Wechsler Memory Scale-Revised Logical Memory II (WMS-R LM II) [27] and Montreal Cognitive Assessment, Japanese version (MoCA-J) [28] were used for screening, as described in Section 2.3. Age, gender, and education were also recorded as participant characteristics. Blood samples were collected after overnight fasting for analysis of fatty acid and LZ at baseline, week 12, and week 24. Neuropsychological tests were performed, and the amount of fatty acids taken from the diet was measured at baseline, 12, and 24 weeks. Each participant filled out the diary for recording supplemental intake and checking that there were no major changes in lifestyle. The Aisei Hospital Ueno Clinic Research Ethics Committee confirmed the principles outlined in the Declaration of Helsinki and approved the protocol (#190411-1). This trial was registered in the University Hospital Medical Information Network (UMIN) Clinical Trial Registry (UMIN000036488) on 12 April 2019. Written informed consent was obtained from all the participants. This report follows the Consolidated Standards of Reporting Trials (CONSORT) statement [29].

### 2.2. Study Design of Trial 2

A randomized, double-blind, placebo-controlled, parallel-group trial was designed to perform a confirmatory evaluation of the effect of supplementation with LCPUFAs + LZ on memory function in healthy older Japanese individuals with memory complaints without dementia. This trial was conducted between August 2021 and October 2022 at medical institutions in Meguro-ku, Tokyo, and in Osaka-shi, Osaka, Japan. A total of 254 participants were recruited from Tokyo, Osaka, and neighboring regions, of whom 192 were enrolled and randomly allocated to two groups: (1) a placebo group, receiving a placebo as a food supplement; (2) an LCPUFAs + LZ group, receiving a food supplement consisting of LCPUFAs (containing 120 mg ARA, 300 mg DHA, and 100 mg EPA per day) combined with LZ (containing 10 mg lutein and 2 mg zeaxanthin per day). The intervention period was set at 12 weeks, because efficacy was found to be exploratory at 12 weeks in Trial 1. Screening and outcome assessments were performed similarly to those in Trial 1, except that they were performed in a remote environment and blood samples were only collected during screening to avoid the risk of COVID-19 infection. The Medical Station Clinic Research Ethics Committee confirmed the principles outlined in the Declaration of Helsinki and approved the protocol (#210708-1). This trial was registered in the University Hospital Medical Information Network (UMIN) Clinical Trial Registry (UMIN000044844) on 13 July 2021. Written informed consent was obtained from all the participants. This report follows the Consolidated Standards of Reporting Trials (CONSORT) statement [29].

### 2.3. Participants in Trials 1 and 2

Both trials included healthy Japanese participants aged 55–79 years who had memory complaints but no dementia, based on a MoCA-J score of more than 17. This MoCA-J criterion corresponds to a Mini-Mental State Examination (MMSE) score of more than 24, the most widely used screening tool for dementia. The MoCA-J criterion was calculated based on the conversion table between MMSE and MoCA scores [30]. The exclusion criteria comprised the following: hearing loss; color blindness; weak vision; a history of neurological disorders (including suspicion of having such disorders); postmenopausal syndrome or hormone therapy; higher memory scores than the benchmark for middle-aged Japanese people (WMS-R LM II > 20); heavy drinking; heavy smoking; irregular lifestyle; history of neuropsychological testing one year prior to each study; allergies to the experimental supplements; and intake of supplements or drugs that affect brain function or lipid metabolism and may therefore influence efficacy evaluations.

### 2.4. Experimental Supplements

The experimental supplements contained purified safflower (High-Linoleic Safflower Oil; The Nisshin OilliO Group, Ltd., Tokyo, Japan) and olive oils (Olive oil RR; Summit oil mill, Chiba, Japan) or LCPUFAs + LZ-containing oils. LCPUFAs + LZ-containing oils were prepared by mixing ARA-containing oil (SUNTGA40S; Nissui Corporation, Tokyo, Japan), DHA/EPA-containing oil (DD-oil; Nissui Corporation, Tokyo, Japan) and LZ-containing oil (FloraGLO; DSM Japan K.K., Tokyo, Japan). Supplementary Tables S1 and S2 show the fatty acid composition of these supplements for each trial. The LCPUFAs + LZ group received LCPUFAs + LZ-containing oil (1560 mg/day in Trial 1 and 1440 mg/day in Trial 2) in 6 soft gelatin capsules, in which 120 mg ARA, 300 mg DHA, 100 mg EPA, 10 mg lutein, and 2 mg zeaxanthin were administered as free body equivalents. The doses of LCPUFAs and LZ were set based on doses that have been found to have effects on cognitive function in previous reports [21,31]. The placebo group received equal amounts of purified safflower and olive oil. Capsules provided to participants in each group were of the same size, color, and flavor. Each participant’s compliance with capsule intake was verified by checking the diary in both trials.

### 2.5. Outcome Assessments

The primary outcome in Trials 1 and 2 was the change in the composite memory score obtained by participants in Cognitrax tests (see Section 2.6). The secondary outcomes were changes in verbal and visual memory as scored in Cognitrax. As the standard dietary intake of LCPUFAs could influence the effect of the experimental supplements, we assessed dietary LCPUFAs intake at baseline, week 12, and week 24 in Trial 1, and at baseline and week 12 in Trial 2. We also assessed the LCPUFAs content in plasma phospholipids at baseline, week 12, and week 24 in Trial 1, and at baseline in Trial 2. In Trial 1, serum LZ levels were also evaluated at baseline, week 12, and week 24. The safety was evaluated according to the incidence of side and/or adverse events during the intervention period. Other psychological evaluations, such as the 36-Item Short Form Health Survey (SF36), were also performed. However, these will be reported elsewhere because the focus of this paper is the combined analysis of memory function.

### 2.6. Memory Function Assessments

We assessed verbal and visual memory tests in Cognitrax, a computerized cognitive function test based on an assessment developed by CNS Vital Signs, Inc. (Morrisville, NC, USA) [32] to evaluate the effects on memory functions. The composite memory score was calculated by summing the verbal and visual memory scores. The outline of each task was as follows:

1. Verbal memory: First, the participants learned 15 words. Immediately after that, from the 30 words (15 learned and 15 unlearned words) that were randomly presented, participants recognized the words that were previously learned. After 30 min or more, again, from the 30 words (15 learned and 15 unlearned words) that were randomly presented, participants recognized the words learned previously. The number of correct responses to 60 trials, including immediate and delayed recognition, was calculated as a verbal memory score.
2. Visual memory: First, participants learned 15 meaningless visual images. Immediately after that, from the 30 images (15 learned and 15 unlearned images) that were randomly presented, participants recognized the images that were previously learned. After 30 min or more, again, from the 30 images (15 learned and 15 unlearned images) that were randomly presented, participants recognized previously learned images. The number of correct responses to 60 trials, including immediate and delayed recognition, was calculated as a visual memory score.

### 2.7. Dietary Assessment and Study Diary

In both trials, brief-type self-administered diet history questionnaires (BDHQ) were performed to estimate dietary intake, such as ARA, DHA, EPA, and their precursors, linoleic and α-linolenic acid, according to a previous study [31]. This estimation was performed by using the Standard Tables of Food Composition in Japan 2010. Participants were instructed to keep records in their diaries for the duration of the study to record supplemental intake and confirm that there were no major lifestyle changes.

### 2.8. Fatty Acid Analysis

In both trials, after blood collection, samples were centrifuged at 2200× *g* for 10 min at 4 °C to separate the plasma and then stored at −80 °C until analysis. Lipids were extracted and purified from the plasma according to the method of the previous report [33], and then thin-layer chromatography (hexane:ether = 7:3) was used to separate the phospholipid fraction. After adding pentadecanoic acid as an internal standard, this fraction was incubated in methanolic HCl to methylate fatty acids at 50 °C for 3 h. The composition of each fatty acid was analyzed by gas–liquid chromatography (Agilent 7890B; Agilent Technologies, Santa Clara, CA, USA) and expressed as a percentage of total fatty acids as described previously [31].

### 2.9. Lutein and Zeaxanthin Analysis

In Trial 1, blood samples were placed at room temperature and centrifuged at 2200× *g* for 10 min at 4 °C to separate the serum, and then stored at −80 °C until analysis. LZ serum concentrations were measured by Kyoto Microbio Laboratory (Kyoto, Japan) using high-performance liquid chromatography as previously described [34].

### 2.10. Sample Size

In Trial 1, referring to previous studies that evaluated the effects of food components on memory function, the number of participants for per protocol set (PPS) analysis was set at 30 in each group, which is the number required for an exploratory study of the effect on memory function. Based on a 25% dropout rate, it was calculated that 40 participants were necessary for each group.

In Trial 2, referring to the results of Trial 1, the number of participants for PPS analysis was set at 79 in each group, which is the number required for a confirmatory study. Based on an 18% dropout rate, it was calculated that 96 participants were necessary for each group. The 79 participants in each group provided a statistical power of 80% at a 5% significance level for analyses to detect differences between groups.

### 2.11. Randomization, Allocation, and Blinding

In Trial 1, the enrolled participants were randomly assigned to the three test groups using the dynamic minimization method and in a 1:1:1 ratio. Age, sex, composite memory score obtained via Cognitrax and composition of ARA and DHA in plasma phospholipids were used as allocation factors to maintain a balance between groups. In Trial 2, allocations were performed in the same manner at a 1:1 ratio to achieve a balance between the groups regarding age, sex, verbal, and visual memory scores obtained via Cognitrax, the composition of ARA and DHA in plasma total lipids, and residential area. A third-party allocation agency managed the allocation information and ensured that double blindness was maintained until the data were fixed in both trials.

### 2.12. Statistical Analysis

The combined analyses of the two trials of composite memory for PPS and those for the subgroup with cognitive decline were performed as the main efficacy assessments. In clinical settings, there is a cutoff value of 25/26 for the MoCA-J for possible MCI [28]. Although this cutoff value has been adopted in clinical settings where sensitivity should be emphasized to avoid the risk of judging a person with possible MCI as healthy, it was also reported to be too high for community-dwelling people [35,36]. In addition, meta-analysis has shown that a value of 22/23 is more appropriate [37], and thus, this cutoff value was used in previous studies [38] for selecting participants with cognitive decline. Hence, in the present study, participants with a MoCA-J score <23 were defined as exhibiting cognitive decline. This value of 23 is consistent with the median value reported in a multiregional cohort study of older community-dwelling Japanese participants [39]. The combined analysis was performed using a random-effects model (DerSimonian and Laird method). Means and standard errors for the combined analysis were the least squares means and standard errors of the change after 12 weeks of intervention, respectively, obtained by analysis of covariance with the pre-intervention values as covariates. The effect size is shown as the mean difference. A similar combined analysis was performed for verbal and visual memory as well as for composite memory as a secondary outcome. These analyses were performed in R v4.2.2 [40] using the “meta” package [41]. All tests were two-sided, and an alpha-level of 0.05 was considered statistically significant. The combined analyses were registered in the UMIN Clinical Trial Registry (UMIN000050451) on 28 February 2023.

In both trials, PPS and subgroup analyses with cognitive decline (MoCA-J score < 23) were performed for efficacy assessments. For quantitative variables, an unpaired Student’s *t*-test was used to compare baseline data between groups. For qualitative variables, a chi-square test was used. A paired *t*-test was used to compare the change from baseline to 12 or 24 weeks after the intervention in each group. An unpaired Student’s *t*-test was used to compare the changes between groups. In addition, an analysis of covariance by baseline memory scores was also performed, as the scores for baseline memory function strongly affected the memory score changes. The full analysis set (FAS) was used for the safety assessment. A chi-square test was used to compare the incidence of adverse events between groups. Although the assessments in Trial 1 were performed for three groups and at three points, *t*-tests and chi-square tests for two groups were used in the analysis without considering multiplicity since it was an exploratory study. IBM SPSS Statistics (IBM Corp., Armonk, NY, USA), R v4.2.2 [40] and Microsoft Excel 365 version 1912–2208 (Washington, DC, USA) were used for analyses. Data are presented as the mean ± standard error (SE). For all statistical analyses, an alpha level of 0.05 was deemed statistically significant, and tests were two-sided.

## 3. Results

### 3.1. Trial 1

#### 3.1.1. Flow of the Participants and Baseline Characteristics in Trial 1

**Figure 1:**
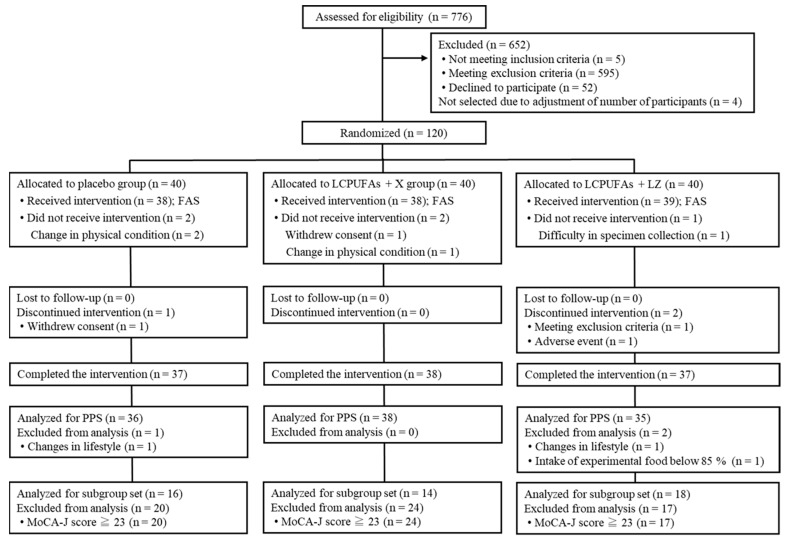
Participant flow of Trial 1. LCPUFAs, long-chain polyunsaturated fatty acids; LZ, lutein and zeaxanthin; MoCA-J, Montreal Cognitive Assessment Japanese version; PPS, per-protocol set; FAS, full analysis set.

The Trial 1 flow diagram of the participants is shown in Figure 1. We screened 776 potential participants, and 120 participants were enrolled and then randomly allocated to the 3 test groups (*n =* 40 per group). A total of 112 participants (placebo: *n =* 37; LCPUFAs + X: *n =* 38; LCPUFAs + LZ: *n =* 37) completed the intervention period, and data from 109 PPS participants (placebo: *n =* 36; LCPUFAs + X: *n =* 38; LCPUFAs + LZ: *n =* 35) were used for the efficacy assessment. Here, only the results of the PPS population are presented because its number (*n =* 109) was almost similar to that of participants who completed all tests (*n =* 112). There were 48 participants in the subgroup analysis with cognitive decline (placebo: *n =* 16; LCPUFAs + X: *n =* 14; LCPUFAs + LZ: *n =* 18), excluding 61 participants with MoCA-J scores of 23 or higher. Composite, verbal, and visual memory tests could not be performed properly in 6 participants (placebo: *n =* 2; LCPUFAs + X: *n =* 3; LCPUFAs + LZ: *n =* 1) and 1 participant (LCPUFAs + LZ: *n =* 1) of the PPS population at week 12 and 24, respectively, and BDHQ, fatty acid analysis, and LZ analysis were not applied for 2 participants (placebo: *n =* 1; LCPUFAs + LZ: *n =* 1) and 1 participant (LCPUFAs + LZ: *n =* 1) at week 12 and 24, respectively; therefore, their scores were treated as missing values. The results of the LCPUFAs + X group are not described below, as this study was concerned with the effectiveness of LCPUFAs + LZ on memory functions. The mean capsule intake within the PPS population was more than 99% in both groups (placebo: 99.4 ± 0.2%; LCPUFAs + LZ: 99.3 ± 0.3%) and not significant between groups (*p* = 0.797). The baseline characteristics, such as age, sex, body mass index (BMI), education, MoCA-J scores, composite, verbal, and visual memory scores, and composition of LCPUFAs (ARA, DHA, and EPA) in plasma phospholipids were matched between groups (Table 1).

#### 3.1.2. PPS Analysis in Trial 1

Supplementary Table S3 shows the dietary intake of fatty acids. The changes in ARA, DHA, EPA, and their precursors, linoleic and α-linolenic acid intake were not different between groups.

Supplementary Table S4 shows the compositions of fatty acids in plasma phospholipids. The compositions of ARA, DHA, and EPA at baseline were not different between groups. The compositions of ARA, DHA, and EPA in the LCPUFAs + LZ group at weeks 12 and 24 were significantly increased (ARA and DHA: *p*  <  0.01, EPA: *p*  <  0.05 vs. baseline). Changes in the compositions of ARA and DHA from baseline to week 12 were significantly different (ARA: *p*  <  0.05, DHA: *p*  <  0.01 vs. placebo) between groups. Changes in the compositions of DHA from baseline to week 24 were also significantly different (*p*  <  0.01 vs. placebo) between groups. Changes in some other fatty acids showed significant differences between groups, but these differences were not physiologically meaningful.

LZ concentrations in serum are shown in Supplementary Table S5. The baseline LZ concentrations were not different between groups. LZ concentrations in the LCPUFAs + LZ group at weeks 12 and 24 were significantly increased (*p*  <  0.01 vs. baseline). Changes in LZ concentrations from baseline to week 12 and week 24 were significantly different (lutein: *p*  <  0.01, zeaxanthin: *p*  <  0.05 vs. placebo) between groups.

Table 2 and Supplementary Table S6 show the composite, verbal, and visual memory scores of each group. No significant differences between groups were detected for any of the baseline scores. In the LCPUFAs + LZ group, changes (Δ adjusted) in the verbal memory score at week 12 were significantly larger than in the placebo group (LCPUFAs + LZ: +1.8, placebo: −0.4). Differences in other memory function scores were consistently larger, but not significantly different, in the LCPUFAs + LZ group than in the placebo group. The results for the composite memory did not change when adjusted for age, gender, educational history, or alcohol intake, respectively.

#### 3.1.3. Subgroup Analysis of Participants with Cognitive Decline in Trial 1

A subgroup analysis was conducted on data from the 34 participants with MoCA-J scores of less than 23 (placebo: *n =* 16; LCPUFAs + LZ: *n =* 18). The results of the PPS analysis suggested the possibility that the power of detection for effect may have decreased at week 24 because 3 repeats of the same memory test may have resulted in a learning effect; however, the effect was in the same direction as that observed at week 12. Therefore, this subgroup analysis focused on the tests performed at week 12. Among these tests, composite, verbal, and visual memory tests could not be performed properly in 2 participants (placebo: *n =* 2) at week 12, and BDHQ, fatty acid analysis, and LZ analysis were not applied for 1 participant (placebo: *n =* 1) at week 12; thus, their scores were treated as missing values. Supplementary Table S7 shows the baseline characteristics. No significant differences between groups were detected for any of the baseline factors.

Supplementary Table S8 shows the dietary intake of fatty acids. No significant differences between groups were detected for the changes in the intake of ARA, DHA, EPA, and their precursors, linoleic and α-linolenic acid between groups.

The compositions of fatty acids in plasma phospholipids for subgroup analysis are shown in Supplementary Table S9. The compositions of ARA, DHA, and EPA at baseline were not different between groups. The compositions of ARA and DHA in the LCPUFAs + LZ group at week 12 were significantly increased (*p*  <  0.01 vs. baseline). Changes in the compositions of ARA, DHA, and EPA from baseline to week 12 were significantly different (*p*  <  0.01 for ARA and DHA, *p*  <  0.05 for EPA vs. placebo) between groups.

LZ concentrations in serum for subgroup analysis are shown in Supplementary Table S10. The baseline LZ concentrations were not different between groups. LZ concentrations in the LCPUFAs + LZ group at week 12 were significantly increased (*p*  <  0.01 vs. baseline). Changes in the L concentrations from baseline to week 12 were significantly different (*p*  <  0.01 vs. placebo) between groups.

**Figure 2:**
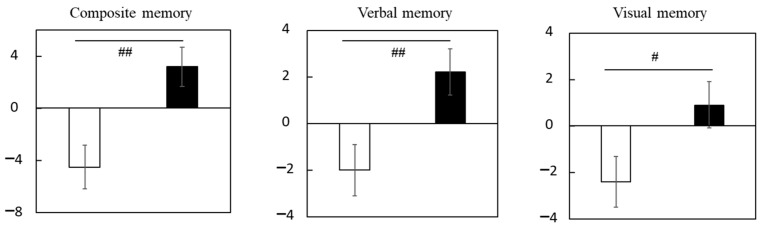
Changes in the scores of episodic memory function tests during the Trial 1 intervention at week 12 in the subgroup with cognitive decline. Values are adjusted by baseline scores and given as the mean ± SE. White column, placebo (*n* = 14); black column, LCPUFAs + LZ (*n* = 18). # *p* < 0.05 and ## *p* < 0.01 vs. placebo group (analysis of covariance by baseline score). LCPUFAs, long-chain polyunsaturated fatty acids; LZ, lutein and zeaxanthin.

Supplementary Table S11 shows the scores of composite, verbal, and visual memory. No significant differences between groups were detected for baseline scores. Changes (Δ adjusted) in composite, verbal, and visual memory scores were significantly larger in the LCPUFAs + LZ group (composite memory: +3.2; verbal memory: +2.2; visual memory: +0.9) than in the placebo group (composite memory: −4.5; verbal memory: −2.0; visual memory: −2.4) (Figure 2, Supplementary Table S11). The results for the composite memory did not change when adjusted for age, gender, educational history, or alcohol intake, respectively.

#### 3.1.4. Safety in Trial 1

No adverse events associated with the supplementation of LCPUFAs + LZ were observed in the FAS population (*n =* 77 in placebo and LCPUFAs + LZ groups). The incidence of adverse events was not different (*p* = 0.569) between the groups (placebo: 52.6%; LCPUFAs + LZ: 46.2%).

### 3.2. Trial 2

#### 3.2.1. Flow of the Participants and Baseline Characteristics in Trial 2

**Figure 3:**
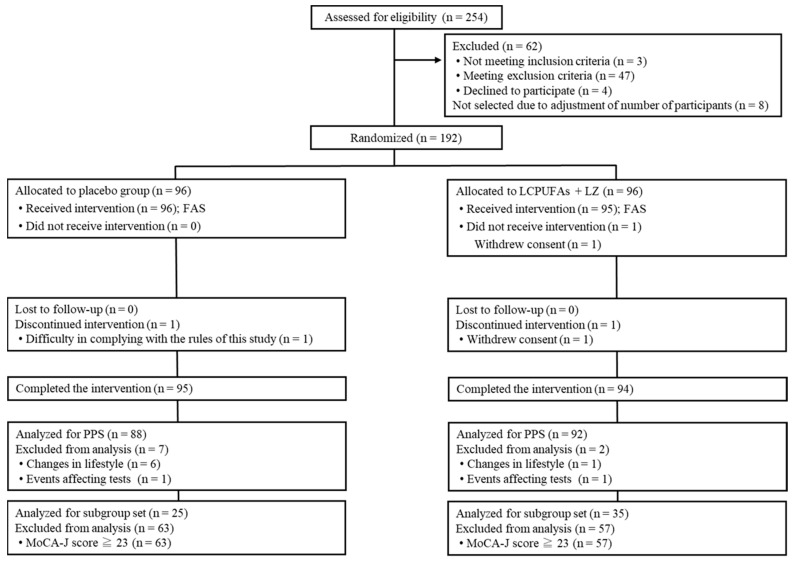
Participant flow of Trial 2. LCPUFAs, long-chain polyunsaturated fatty acids; LZ, lutein and zeaxanthin; MoCA-J, Montreal Cognitive Assessment Japanese version; PPS, per-protocol set; FAS, full analysis set.

The Trial 2 flow diagram of the participants is shown in Figure 3. We screened 254 potential participants, among whom 192 participants were enrolled and randomly allocated to the test groups (*n =* 96 per group). A total of 189 participants (placebo: *n =* 95; LCPUFAs + LZ: *n =* 94) completed the 12-week intervention period, and data from 180 PPS participants (placebo: *n =* 88; LCPUFAs + LZ: *n =* 92) were used for the efficacy assessment. Here, only the results of PPS population are presented because the number of the PPS population (*n =* 180) was almost similar to that of participants who completed all tests (*n =* 189). There were 60 participants in the subgroup analysis with cognitive decline (placebo: *n =* 25; LCPUFAs + LZ: *n =* 35), excluding 120 participants with MoCA-J scores of 23 or higher. In the PPS population, it was not possible to properly perform composite memory tests at week 12 for 5 participants (placebo: *n =* 3; LCPUFAs + LZ: *n =* 2), verbal memory tests at week 12 for 4 participants (placebo: *n =* 3; LCPUFAs + LZ: *n =* 1), visual memory tests at week 12 for 2 participants (placebo: *n =* 1; LCPUFAs + LZ: *n =* 1), and BDHQ at baseline for 1 participant (LCPUFAs + LZ: *n =* 1) and at week 12 for 2 participants (placebo: *n =* 1; LCPUFAs + LZ: *n =* 1); therefore, these scores were treated as missing values. The mean capsule intake within the PPS population was more than 99% in both groups (placebo: 99.6 ± 0.2%; LCPUFAs + LZ: 99.6 ± 0.2%) and not significant between groups (*p* = 0.860). The baseline characteristics, such as age, sex, residential area, BMI, education, MoCA-J scores, composite, verbal, and visual memory scores, and composition of LCPUFAs (ARA, DHA, and EPA) in plasma phospholipids were matched between groups (Table 3).

#### 3.2.2. PPS Analysis in Trial 2

Supplementary Table S12 shows the dietary intake of fatty acids. The changes in ARA, DHA, EPA, and their precursors, linoleic and α-linolenic acid intake were not different between groups.

Table 4 shows the composite, verbal, and visual memory scores of each group. No significant differences between groups were detected for any of the baseline scores. No significant differences were detected in the changes in the composite, verbal, and visual memory scores. The results for the composite memory did not change when adjusted for age, gender, educational history, or alcohol intake, respectively.

#### 3.2.3. Subgroup Analysis of Participants with Cognitive Decline in Trial 2

A subgroup analysis was conducted on data from the 60 participants with MoCA-J scores of less than 23 (placebo: *n =* 25; LCPUFAs + LZ: *n =* 35). Among the tests performed, composite memory tests at week 12 for 2 participants (placebo: *n* = 1; LCPUFAs + LZ: *n* = 1), verbal memory tests at week 12 for 2 participants (placebo: *n* = 1; LCPUFAs + LZ: n = 1), and BDHQ at baseline for 1 participant (LCPUFAs + LZ: *n* = 1) and at week 12 for 1 participant (placebo: *n* = 1); therefore, their scores were treated as missing values. Supplementary Table S13 shows the baseline characteristics. No significant differences between groups were detected for any of the baseline factors.

Supplementary Table S14 shows the dietary intake of fatty acids. No significant differences between groups were detected for the changes in the intake of ARA, DHA, EPA, and their precursors, linoleic and α-linolenic acid between groups.

**Figure 4:**
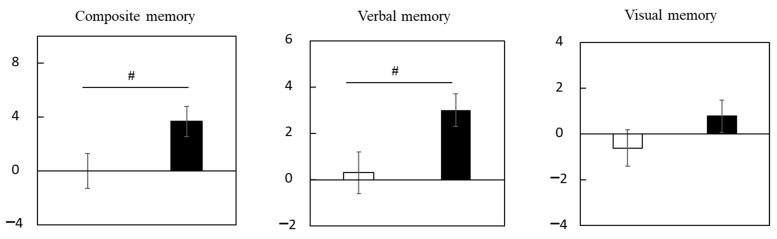
Changes in the scores of episodic memory function tests during the Trial 2 intervention at week 12 in the subgroup with cognitive decline. Values are adjusted by baseline scores and given as the mean ± SE. White column, placebo (*n* = 24 for composite and verbal memory, *n* = 25 for visual memory); black column, LCPUFAs + LZ (*n* = 34 for composite and verbal memory, *n* = 35 for visual memory). # *p* < 0.05 vs. placebo group (analysis of covariance by baseline score). LCPUFAs, long-chain polyunsaturated fatty acids; LZ, lutein and zeaxanthin.

Supplementary Table S15 shows the scores of composite, verbal, and visual memory. No significant differences between groups were detected for baseline scores. Changes (Δ adjusted) in composite and verbal memory scores were significantly larger in the LCPUFAs + LZ group (composite memory: +3.7; verbal memory: +3.0) than in the placebo group (composite memory: +0.0; verbal memory: +0.3). Changes (Δ adjusted) in visual memory scores were also larger, although not significant, in the LCPUFAs + LZ (visual memory: +0.8) group than in the placebo group (visual memory: −0.6) (Figure 4, Supplementary Table S15). The results for the composite memory did not change when adjusted for age, gender, educational history, or alcohol intake, respectively.

#### 3.2.4. Safety in Trial 2

No adverse events associated with the supplementation of LCPUFAs + LZ were observed in the FAS population (*n =* 191). The incidence of adverse events was not different (*p* = 0.050) between the groups (placebo: 31.3%; LCPUFAs + LZ: 18.9%).

### 3.3. Combined Analysis of the Two Trials

**Figure 5:**
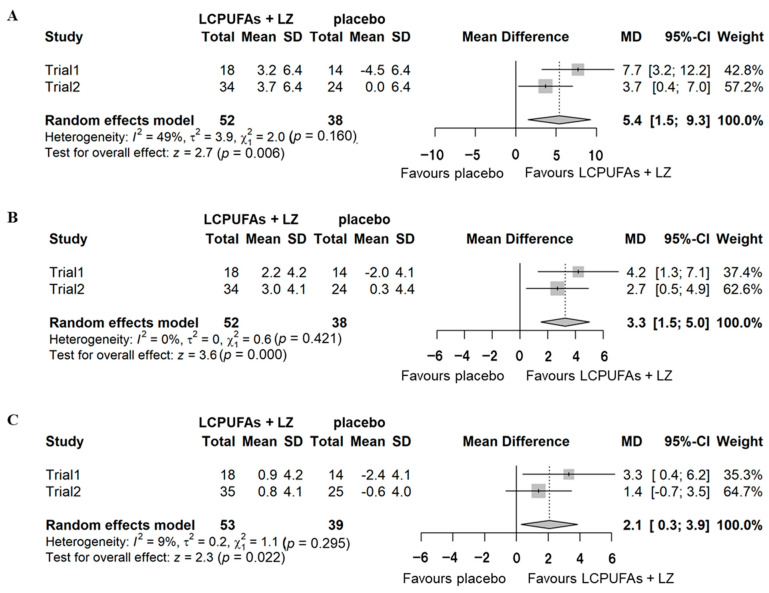
Combined analysis of the changes (Δ-adjusted) in the scores of episodic memory function tests for subgroups with cognitive decline in Trials 1 and 2. (**A**) composite memory; (**B**), verbal memory; (**C**), visual memory. LCPUFAs, long-chain polyunsaturated fatty acids; LZ, lutein and zeaxanthin; SD, standard deviation; MD, mean difference; CI, confidence interval.

A combined analysis was performed based on the means and standard deviations of the results of the two trials. Supplementary Figure S1 shows the results of the combined PPS analysis. No significant effects were detected on composite (mean difference = 1.2 [95% CI: −1.5–4.0], *p* = 0.374), verbal (mean difference = 1.2 [95% CI: −0.4–2.8], *p* = 0.149), and visual memory (mean difference = −0.1 [95% CI: −1.1–0.9], *p* = 0.805) scores. The results of the combined subgroup analysis with cognitive decline are shown in Figure 5. Significant effects were detected on composite (mean difference = 5.4 [95% CI: 1.5–9.3], *p* = 0.006), verbal (mean difference = 3.3 [95% CI: 1.5–5.0], *p* < 0.001), and visual memory (mean difference = 2.1 [95% CI: 0.3–3.9], *p* = 0.022) scores.

## 4. Discussion

In the present study, we conducted a combined analysis of two trials to evaluate the effects of LCPUFAs + LZ supplementation on memory function in healthy older Japanese individuals with memory complaints without dementia. There were no significant differences between groups in the combined PPS analysis. However, significant improvements were observed in the combined analysis of the subgroup with cognitive decline. This study suggests for the first time that the combined intake of LCPUFAs and LZ could potentially improve memory function in healthy older Japanese individuals with cognitive decline without dementia.

In both trials, the cognitive function of the participants was considered to be at the same level as that of the general older population, as the average MoCA-J scores obtained at baseline (Table 1 and Table 3) were at about the same level as the average scores previously reported for older community-dwelling Japanese people [39]. In terms of LCPUFAs, ARA (9.3% in Trial 1, 9.9% in Trial 2), DHA (6.5% in Trial 1, 6.7% in Trial 2), and EPA (1.9% in Trial 1, 2.0% in Trial 2) compositions in plasma phospholipids and dietary ARA (Trial 1: 172 mg/day, Trial 2: 178 mg/day), DHA (Trial 1: 482 mg/day, Trial 2: 498 mg/day), and EPA (Trial 1: 272 mg/day, Trial 2: 285 mg/day) intakes were within the range reported by previous studies on older Japanese people [31,42,43,44,45,46]. These results suggest that the participants in both studies corresponded well with the general population of older Japanese.

In the PPS analysis in Trial 2 of our study, which was a confirmatory study, no significant changes were noted in episodic memory scores. In Trial 2, in which the number of cases was designed based on the positive changes observed in Trial 1, no significant efficacy of LCPUFAs + LZ was detected. One factor for which no significant difference was detected was the difference between the two trials in the distribution of the baseline cognitive function scores. In Trial 1, the median MoCA-J score was 23, and the percentage of those with a score below 23 was 44%. In contrast, the median score in Trial 2 was 24, and the percentage of participants with a score below 23 was 33%, indicating that the participants in Trial 2 had higher cognitive function. In previous reports, food components were found to have a greater impact on the memory function of participants with lower cognitive function [5,6,23,24]. Consistent with this trend, no effect was detected in Trial 2, which comprised participants with relatively high cognitive function. Further studies are needed to determine the effect of LCPUFAs + LZ on memory function in healthy older Japanese individuals with memory complaints without dementia.

In the present study, the combined analysis of the subgroup with cognitive decline, LCPUFAs + LZ supplementation resulted in a significant improvement of composite memory. For the composite memory scores in Cognitrax, the average score for subjects 60–69 years old was 94.6, and 90.7 for those 70–79 years old, reflecting a decrease of 3.9 points over the decade [32]. The change observed in the combined analysis for the subgroup with cognitive decline in this study was 5.4, which is more than the 10-year change described above, and thus is a physiologically meaningful level. Improvements were also observed in verbal and visual memory, consistent with the results for composite memory. The number of participants required to confirm the efficacy of the supplementation, as calculated based on the results of the subgroup analysis in Trial 2, was approximately 100. The total number of participants in the combined analysis was 90, approximately the same as the requirement, which contributed to improving the statistical power of the analyses performed compared with the individual trials.

The improvement in visual memory was less than verbal memory for both trials. In addition, several other intervention studies using Cognitrax [47,48,49] revealed that the effectiveness of Cognitrax on verbal memory was easier to detect than visual memory. Cognitrax distinguishes verbal memory by requiring the memorization of “meaningful” words, which may be easier than visual memory, which requires the memorization of “meaningless” shapes. One possibility is that differences in difficulty influenced the ability to detect validity. However, the present study does not provide an accurate comparison of the differences in difficulty, and further research is required, to compare the power to detect validity for the memory of “meaningful” and “meaningless” shapes.

Furthermore, when setting the threshold for MoCA-J scores in the subgroup analysis at 22 and 21 points (rather than 23), significant improvements were consistently observed in the combined analysis. Additionally, no significant differences were detected for composite memory in the combined PPS analysis or subgroups with MoCA-J scores ≥23. These results suggest that LCPUFAs + LZ intake helps improve memory function in healthy older individuals with cognitive decline, thereby supporting Hypothesis 2: Memory function is more likely to improve by LCPUFAs + LZ in participants with cognitive decline than in participants with normal cognitive function. These subgroup analyses based on baseline cognitive status are important for clarifying who may benefit from the intervention, which can pave the way for more efficient treatment strategies.

In this study, the PPS analysis found that there was no efficacy on memory function, which is consistent with previous studies. For example, Dangour et al. found that DHA (500 mg/day) and EPA (200 mg/day) for 24 months had no effect on memory function in cognitively normal older people [23]. In addition, van de Rest et al. reported that a 26-week intake of DHA (847 mg/day) and EPA (1093 mg/day) had no effect on memory function in cognitively normal older people [24]. In the present study, the DHA and EPA doses were 300 mg/day and 100 mg/day, respectively, which were lower than those in the studies mentioned above. Furthermore, the duration was shorter than in previous studies, so the lack of efficacy in the PPS analysis in this study is consistent with their findings.

Improvements in some memory function test items were seen in a study in which DHA 800 mg/day and L 12 mg/day were consumed for four months [50], seemingly contradicting the present study. However, they did not observe improvement in the number of correct answers in word list and shopping list recalls, which is consistent with similar measures in the present study. Since improvements in the number of times it took to recall the list were discovered, it is possible that the differences in the indices influenced the results.

The present study found efficacy on memory function in healthy older Japanese individuals with cognitive decline without dementia, which is consistent with previous research. For example, Yurko-Mauro et al. reported that 24 weeks of DHA (900 mg/day) improved memory function in healthy subjects with age-related cognitive decline [5]. In addition, Lee et al. found that 12 months of DHA (1300 mg/day) and EPA (450 mg/day) improved memory function in healthy subjects with MCI [6]. Thus, the fact that efficacy was obtained in studies using higher doses of DHA and EPA than the present study, supports the results of this study.

According to previous reports, LCPUFA [5,6] and LZ [20] have the potential to improve memory. However, reports on ARA, DHA, or EPA have not found improvement in memory function at doses as low as those used in this study, and these alone are unlikely to be effective in improving memory function. Furthermore, the efficacy of LZ at the same dose used in the present study was not confirmed in a study of people with low cognitive function [21]. Based on the above, it is unlikely that LCPUFA or LZ would contribute to the effectiveness of memory function on their own, rather, it is likely that both contributed to the effectiveness.

Synaptic plasticity, which is necessary for memory function and declines with age, can be improved by the intake of ARA, DHA, or EPA [51,52,53]. In addition, it has been reported that synaptic plasticity is improved by the following mechanisms. DHA and EPA improve the age-related decrease in synaptic membrane fluidity [54], and ARA improves the decrease in neuronal membrane fluidity [55]. DHA and EPA ameliorate age-related decreases in the expression of N-methyl-D-aspartate neuroreceptors [56] and increase brain-derived neurotrophic factor [57,58]. ARA and DHA have also been reported to ameliorate age-related decreases in neurogenesis [4,59]. LZ are carotenoids present in the brain [15] that possess anti-inflammatory and antioxidant properties. In terms of antioxidant effects, the amount of DHA oxide, neuroprostanes, is inversely correlated with the amount of L in mitochondria of the frontal cortex and striatum [60], and LZ suppresses the increase in mitochondrial reactive oxygen species in cultured neural cells [61]. In terms of anti-inflammatory effects, L suppresses increased inflammation in cultured microglia [62] and LZ supplementation decreases CRP levels, a marker of inflammation, in human blood [63]. These antioxidant and anti-inflammatory effects can be expected to contribute to the improvement of synaptic plasticity [64,65,66,67,68] and neuronal protection [17,67,69,70], contributing to improved memory function. Moreover, oxidative and inflammatory markers in the blood are negatively correlated with cognitive function in older people [71], and oxidative and inflammatory status may increase in those with cognitive decline. Given this negative correlation and the anti-inflammatory and antioxidant effects of LZ, it is reasonable that the efficacy of LCPUFAs + LZ was observed in healthy older individuals with cognitive decline who may be experiencing increased inflammation and oxidation levels. In the future, changes in inflammatory and oxidative markers in the blood induced by LCPUFAs + LZ supplementation should be evaluated to reveal the underlying mechanisms of its effects.

This study has several strengths. No adverse events associated with the supplementation of LCPUFAs + LZ were observed, indicating that LCPUFAs + LZ supplementation in both Trials 1 and 2 can be considered safe. In addition, the average intake rates of the capsules in both trials used for the combined analysis were high, at more than 99% each. In Trial 1, significant increases in ARA (+ 0.7% at week 12 and +0.7% at week 24), DHA (+ 1.0% at week 12 and +0.8% at week 24), and EPA (+ 0.4% at week 12 and +0.3% at week 24) compositions in plasma PL, and L (+ 0.20 μg/mL at week 12 and +0.19 μg/mL at week 24), and Z (+ 0.06 μg/mL at week 12 and +0.05 μg/mL at week 24) concentrations in serum were observed in the LCPUFAs + LZ group in Trial 1 (Supplementary Tables S4 and S5). These changes are reasonable and physiologically meaningful as they are within the range reported by previous studies on the effects of supplementation on cognitive function [31]. The lifestyle diary confirmed no major changes, implying there were no significant changes in factors that could affect memory function throughout the study period, such as exercise, eating, and drinking habits. The amount of change in ARA, DHA, and EPA intake, the intervening factors, did not differ significantly between groups during the study period. Although the amount of LZ intake could not be measured due to the limitations of the dietary survey, the average intake of LZ among Japanese has been reported to be 0.35 mg/day [72], which is quite small compared to the amount of LZ supplemented in this study, and dietary LZ intake is not assumed to affect efficacy. These lifestyle-related data indicated that both trials were completed with high compliance among the participants. Furthermore, the effects on memory function observed in the subgroup with cognitive decline were consistent across the composite, verbal, and visual memory tests, with significant differences being detected across combined analyses. Finally, significant effects were detected not only for the specific threshold of 23 points but also when the threshold score was lowered further (22 and 21), supporting our hypothesis that LCPUFAs + LZ is effective in healthy older individuals with cognitive decline.

This study also has three major limitations. First, the data from the combined analysis in which the effect of LCPUFAs + LZ was detected were based on data from a subgroup analysis. Second, the scoring test used in this study, Cognitrax, provides a relatively easy task, and the results may change if the difficulty of the test changes. It is necessary to examine how the use of a relatively difficult test, for example, the Wechsler memory scale of logical memory, would affect the results for subjects other than those with cognitive decline. Third, this study was conducted on Japanese participants, and it is unknown whether similar results would have been obtained if the study had been conducted on people in low-income countries with different educational environment and nutritional status than Japan. However, ARA, DHA, and EPA which are essential fatty acids, and LZ which exist in the human brain, have antioxidant and anti-inflammatory effects, are considered necessary for the human body and brain, regardless of the environment. In order to clarify the efficacy of these components in non-Japanese individuals, it is necessary to conduct studies in other countries.

## 5. Conclusions

No clear effect was detected for LCPUFAs + LZ on memory function in healthy older Japanese individuals with memory complaints without dementia. Further investigation, including evaluation using other memory assessments, is needed to determine the effect of LCPUFAs + LZ as an intervention. In contrast, supplementation with LCPUFAs + LZ improved memory function in healthy older Japanese individuals with cognitive decline without dementia. Future interventional studies based on a detailed understanding of the cognitive function status of the participants at baseline will lead to appropriate judgments of the intervention’s effect on memory function.

## Figures and Tables

**Table 1:** Baseline characteristics of PPS participants in Trial 1.

	Placebo	LCPUFAs + LZ	*p*
Age (years) ^a^	65.0 ± 0.8	65.7 ± 1.0	0.586
Sex (M/F) ^b^	17/19	15/20	0.711
BMI (kg/m^2^) ^a^	22.4 ± 0.4	22.7 ± 0.5	0.682
Education (years) ^a^	13.9 ± 0.3	14.6 ± 0.4	0.157
MoCA-J ^a^	23.3 ± 0.4	22.8 ± 0.5	0.472
Composite memory ^a^	95.2 ± 1.2	95.1 ± 1.0	0.925
Verbal memory ^a^	50.1 ± 0.8	50.8 ± 0.8	0.547
Visual memory ^a^	45.1 ± 0.8	44.3 ± 0.6	0.409
ARA in plasma PL (%) ^a^	9.0 ± 0.2	9.5 ± 0.2	0.159
DHA in plasma PL (%) ^a^	6.5 ± 0.3	6.6 ± 0.2	0.821
EPA in plasma PL (%) ^a^	1.8 ± 0.2	1.9 ± 0.2	0.583

Mean ± SE. Placebo group (*n* = 34 for episodic memory scores, *n* = 36 for all other data). LCPUFAs + LZ group (*n* = 34 for episodic memory scores, *n* = 35 for all other data). For participants with missing values in episodic memory scores at week 12, baseline values were also removed. There was no significant difference between groups in the baseline data (^a^ unpaired Student’s *t*-test, ^b^ chi-square test). PPS, per-protocol set; LCPUFAs, long-chain polyunsaturated fatty acids; LZ, lutein and zeaxanthin; BMI, body mass index; MoCA-J, Montreal Cognitive Assessment Japanese version; ARA, arachidonic acid; PL, phospholipids; DHA, docosahexaenoic acid; EPA, eicosapentaenoic acid.

**Table 2:** Episodic memory tests by group at the baseline and at 12 weeks in Trial 1.

	Group	Baseline	12 Weeks	Δ	Δ Adjusted
**Episodic memory**					
Composite memory	Placebo	95.2 ± 1.2	94.7 ± 1.6	−0.5 ± 1.5	−0.5 ± 1.3
	LCPUFAs + LZ	95.1 ± 1.0	97.7 ± 1.3 *	2.6 ± 1.2	2.6 ± 1.3
Verbal memory	Placebo	50.1 ± 0.8	49.8 ± 1.0	−0.3 ± 0.9	−0.4 ± 0.7
	LCPUFAs + LZ	50.8 ± 0.8	52.5 ± 0.8 *	1.7 ± 0.7	1.8 ± 0.7 #
Visual memory	Placebo	45.1 ± 0.8	44.9 ± 0.9	−0.2 ± 1.0	0.1 ± 0.8
	LCPUFAs + LZ	44.3 ± 0.6	45.3 ± 0.7	1.0 ± 0.9	0.7 ± 0.8

Mean ± SE; Placebo (*n* = 34) and LCPUFAs + LZ (*n* = 34) groups. For participants with missing values in episodic memory scores at week 12, baseline values were also removed. There was no significant difference between groups at the baseline (unpaired Student’s *t*-test). * *p* < 0.05 vs. baseline (paired *t*-test). # *p* < 0.05 vs. placebo (unpaired Student’s *t*-test or analysis of covariance by baseline score). LCPUFAs, long-chain polyunsaturated fatty acids; LZ, lutein and zeaxanthin.

**Table 3:** Baseline characteristics of PPS participants in Trial 2.

	Placebo	LCPUFAs + LZ	*p*
Age (years) ^a^	65.1 ± 0.6	65.1 ± 0.6	0.972
Sex (M/F) ^b^	48/40	48/44	0.749
Residential area (Tokyo/Osaka) ^b^	39/49	41/51	0.973
BMI (kg/m^2^) ^a^	22.8 ± 0.3	22.7 ± 0.3	0.729
Education (years) ^a^	14.5 ± 0.2	14.9 ± 0.2	0.135
MoCA-J ^a^	23.7 ± 0.3	23.8 ± 0.3	0.899
Composite memory ^a^	96.0 ± 0.8	95.9 ± 0.8	0.948
Verbal memory ^a^	50.4 ± 0.5	50.5 ± 0.5	0.938
Visual memory ^a^	45.4 ± 0.5	45.4 ± 0.4	0.980
ARA in plasma PL (%) ^a^	9.8 ± 0.2	9.9 ± 0.2	0.867
DHA in plasma PL (%) ^a^	6.6 ± 0.2	6.8 ± 0.2	0.289
EPA in plasma PL (%) ^a^	2.1 ± 0.2	2.0 ± 0.1	0.392

Mean ± SE; Placebo group (*n* = 85 for composite and verbal memory scores, *n* = 87 for visual memory score, *n* = 88 for all other data). LCPUFAs + LZ group (*n* = 90 for composite memory score, *n* = 91 for verbal and visual memory scores, *n* = 92 for all other data). For participants with missing values in episodic memory scores at week 12, baseline values were also removed. There was no significant difference between groups in the baseline data (^a^ unpaired Student’s *t*-test, ^b^ chi-square test). PPS, per-protocol set; LCPUFAs, long-chain polyunsaturated fatty acids; LZ, lutein and zeaxanthin; BMI, body mass index; MoCA-J, Montreal Cognitive Assessment Japanese version; ARA, arachidonic acid; PL, phospholipids; DHA, docosahexaenoic acid; EPA, eicosapentaenoic acid.

**Table 4:** Episodic memory tests by group during the intervention in Trial 2.

	Group	Baseline	12 Weeks	∆	∆ Adjusted
**Episodic memory**					
Composite memory	Placebo	96.0 ± 0.8	96.3 ± 0.9	0.3 ± 0.8	0.3 ± 0.7
	LCPUFAs + LZ	95.9 ± 0.8	96.4 ± 0.7	0.5 ± 0.8	0.5 ± 0.7
Verbal memory	Placebo	50.4 ± 0.5	51.5 ± 0.5 *	1.1 ± 0.5	1.1 ± 0.5
	LCPUFAs + LZ	50.5 ± 0.5	52.0 ± 0.5 **	1.5 ± 0.6	1.6 ± 0.5
Visual memory	Placebo	45.4 ± 0.5	44.5 ± 0.6	−0.8 ± 0.5	−0.8 ± 0.4
	LCPUFAs + LZ	45.4 ± 0.4	44.3 ± 0.4 *	−1.1 ± 0.5	−1.1 ± 0.4

Mean ± SE. Placebo group (*n* = 85 for composite and verbal memory scores, *n* = 87 for visual memory score). LCPUFAs + LZ group (*n* = 90 for composite memory score, *n* = 91 for verbal and visual memory scores). For participants with missing values in episodic memory scores at week 12, baseline values were also removed. There was no significant difference between groups at the baseline (unpaired Student’s *t*-test). * *p* < 0.05 and ** *p* < 0.01 vs. baseline (paired *t*-test). There was no significant difference in changes (∆) and changes adjusted by baseline (∆ adjusted) between groups (unpaired Student’s *t*-test or analysis of covariance by baseline score). LCPUFAs, long-chain polyunsaturated fatty acids; LZ, lutein and zeaxanthin.

## Data Availability

All data supporting the findings of this study are included in the published article (and its Supplementary Information files).

## Supplementary Materials

The following supporting information can be downloaded at: https://www.mdpi.com/article/10.3390/nu15132825/s1, Table S1: Fatty acid composition of the experimental supplement in Trial 1; Table S2: Fatty acid composition of the experimental supplement in Trial 2; Table S3: Dietary assessment by group during the intervention in Trial 1; Table S4: Fatty acid composition in plasma phospholipid by group during the intervention in Trial 1; Table S5: Lutein and zeaxanthin concentration in serum by group during the intervention in Trial 1; Table S6: Episodic memory tests by group at the baseline and at 24 Weeks in Trial 1; Table S7: Baseline characteristics of participants for subgroup analysis in Trial 1; Table S8: Dietary assessment by group for subgroup analysis during the intervention in Trial 1; Table S9: Fatty acid composition of plasma phospholipid by group for subgroup analysis during the intervention in Trial 1; Table S10: Lutein and zeaxanthin concentration in serum by group for subgroup analysis during the intervention in Trial 1; Table S11: Episodic memory tests by group during the intervention for subgroup analysis in Trial 1; Table S12: Dietary assessment by group during the intervention in Trial 2; Table S13: Baseline characteristics of the participants for subgroup analysis in Trial 2; Table S14: Dietary assessment by group for subgroup analysis during the intervention in Trial 2; Table S15: Episodic memory tests by group during the intervention for subgroup analysis in Trial 2; Figure S1: Combined analysis of the changes (Δ-adjusted) in the scores of episodic memory function tests for per-protocol sets in Trials 1 and 2.
