# Supplementary material for: Effects of Long-Chain Polyunsaturated Fatty Acids in Combination with Lutein and Zeaxanthin on Episodic Memory in Healthy Older Adults
Source: Nutrients. 2023 Jun 21;15(13):2825. doi: 10.3390/nu15132825 (PMC10343771; doi:10.3390/nu15132825)
Supplement: Supplementary file 1 [file nutrients-15-02825-s001.zip › Proof_Figure S1.pdf]

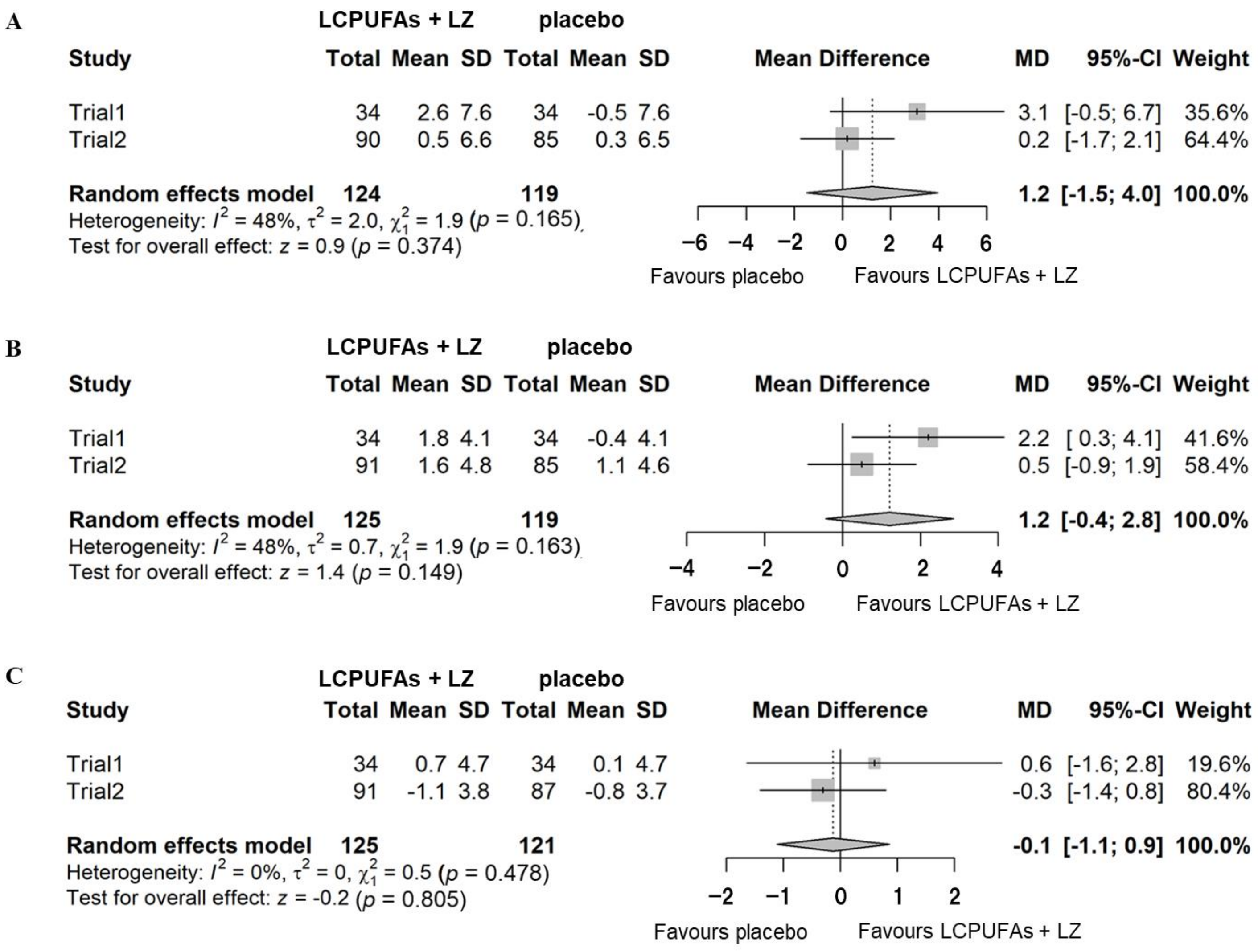

Figure S1. Combined analysis of the changes (⊿-adjusted) in the scores of episodic memory function tests for per-protocol sets in Trials 1 and 2. A, composite memory; B, verbal memory; C, visual memory. LCPUFAs, long-chain polyunsaturated fatty acids; LZ, lutein and zeaxanthin; SD, standard deviation; MD, mean difference; CI, confidence interval.
