# Supplementary material for: Effects of Long-Chain Polyunsaturated Fatty Acids in Combination with Lutein and Zeaxanthin on Episodic Memory in Healthy Older Adults
Source: Nutrients. 2023 Jun 21;15(13):2825. doi: 10.3390/nu15132825 (PMC10343771; doi:10.3390/nu15132825)
Supplement: Supplementary file 1 [file nutrients-15-02825-s001.zip › Proof_supplementary_table.pdf]

Supplementary Table S1. Fatty acid composition of the experimental supplement in Trial 1

Fatty acid		Placebo (%)	LCPUFAs + LZ (%)
Palmitic acid	16:0	14.8	12.1
Stearic acid	18:0	14.6	17.7
Arachidic acid	20:0	0.6	0.7
Behenic acid	22:0	0.2	0.9
Lignoceric acid	24:0	-	1.7
Palmitoleic acid	16:1	0.7	1.8
Oleic acid	18:1	60.5	6.2
Eicosenoic acid	20:1	0.2	1.3
Docosenoic acid	22:1	-	1.0
Tetracosenoic acid	24:1	-	0.4
Linoleic acid	18:2n-6	6.5	3.6
Eicosadienoic acid	20:2n-6	-	0.3
Dihomo-g-linolenic acid	20:3n-6	-	0.8
ARA	20:4n-6	-	9.5
Docosatetraenoic acid	22:4n-6	-	0.3
Docosapentaenoic acid	22:5n-6	-	1.2
α-Linolenic acid	18:3n-3	0.6	0.3
EPA	20:5n-3	-	7.5
Docosapentaenoic acid	22:5n-3	-	1.6
DHA	22:6n-3	-	22.6
Others		1.3	8.5
Total		100.0	100.0

LCPUFAs, long-chain polyunsaturated fatty acids; LZ, lutein and zeaxanthin; ARA, arachidonic acid; DHA, docosahexaenoic acid; EPA, eicosapentaenoic acid.

Supplementary Table S2. Fatty acid composition of the experimental supplement in Trial 2

Fatty acid		Placebo (%)	LCPUFAs + LZ (%)
Palmitic acid	16:0	14.8	11.3
Stearic acid	18:0	13.8	14.8
Arachidic acid	20:0	0.6	0.6
Behenic acid	22:0	0.2	0.8
Lignoceric acid	24:0	-	1.6
Palmitoleic acid	16:1	0.9	1.8
Oleic acid	18:1	58.5	6.4
Eicosenoic acid	20:1	0.2	1.5
Docosenoic acid	22:1	-	1.2
Tetracosenoic acid	24:1	-	0.5
Linoleic acid	18:2n-6	9.5	4.1
Eicosadienoic acid	20:2n-6	-	0.3
Dihomo-g-linolenic acid	20:3n-6	-	1.0
ARA	20:4n-6	-	9.9
Docosatetraenoic acid	22:4n-6	-	0.3
Docosapentaenoic acid	22:5n-6	-	1.4
α-Linolenic acid	18:3n-3	0.5	0.3
EPA	20:5n-3	-	8.0
Docosapentaenoic acid	22:5n-3	-	1.7
DHA	22:6n-3	-	24.7
Others		1.0	7.8
Total		100.0	100.0

LCPUFAs, long-chain polyunsaturated fatty acids; LZ, lutein and zeaxanthin; ARA, arachidonic acid; DHA, docosahexaenoic acid; EPA, eicosapentaenoic acid.

Supplementary Table S3. Dietary assessment by group during the intervention in Trial 1

	Group	Baseline			12 Weeks			Δ (12-Week Baseline)			24 Weeks			Δ (24-Week Baseline)		
Energy (kcal/day)	Placebo	1592	±	68	1527	±	56	− 57	±	48	1535	±	54	−56	±	55
	LCPUFAs + LZ	1660	±	84	1664	±	117	8	±	68	1671	±	87	34	±	50
Carbohydrate (g/day)	Placebo	201	±	10	198	±	9	−1	±	8	201	±	7	−1	±	8
	LCPUFAs + LZ	201	±	11	210	±	13	11	±	9	213	±	11	14	±	8
Protein (g/day)	Placebo	60	±	3	59	±	3	−1	±	2	60	±	3	0	±	2
	LCPUFAs + LZ	67	±	4	65	±	5	−1	±	3	67	±	4	2	±	2
Fat (g/day)	Placebo	53	±	3	49	±	3	−3	±	2	49	±	3	−4	±	2
	LCPUFAs + LZ	58	±	4	56	±	5	−3	±	3	55	±	4	−2	±	2
LA (mg/day)	Placebo	10327	±	566	9792	±	547	−412	±	451	9706	±	535	−621	±	449
	LCPUFAs + LZ	11533	±	740	10668	±	892	−950	±	579	10912	±	809	−467	±	465
ALA (mg/day)	Placebo	1647	±	96	1552	±	93	−80	±	82	1518	±	98	−129	±	82
	LCPUFAs + LZ	1849	±	124	1681	±	151	−178	±	98	1709	±	142	−108	±	83
ARA (mg/day)	Placebo	160	±	9	159	±	10	−1	±	9	165	±	11	6	±	9
	LCPUFAs + LZ	184	±	13	183	±	16	−3	±	10	188	±	13	5	±	7
DHA (mg/day)	Placebo	444	±	45	449	±	43	1	±	40	470	±	41	26	±	36
	LCPUFAs + LZ	522	±	49	485	±	48	−32	±	35	533	±	48	22	±	28
EPA (mg/day)	Placebo	251	±	30	255	±	28	1	±	25	263	±	26	12	±	23
	LCPUFAs + LZ	294	±	30	268	±	29	−20	±	23	296	±	30	11	±	19

Mean ± SE

Placebo group (n = 36 at baseline, n = 35 at week 12, and n = 36 at week 24).

LCPUFAs + LZ group (n = 35 at baseline, n = 34 at week 12, and n = 34 at week 24).

There were no significant differences between groups at the baseline and in change (Δ) (unpaired Student's *t*-test).

There were no significant differences between the baseline and 12-week, and in the baseline and 24-week data in each group (paired *t*-test).

LCPUFAs, long-chain polyunsaturated fatty acids; LZ, lutein and zeaxanthin; LA, linoleic acid; ALA, α-linolenic acid; ARA, arachidonic acid; DHA, docosahexaenoic acid; EPA, eicosapentaenoic acid.

Supplementary Table S4. Fatty acid composition in plasma phospholipid by group during the intervention in Trial 1

	Group	Baseline	12 Weeks		Δ (12-Week Baseline)		24 Weeks		Δ (24-Week Baseline)	
PA (%)	Placebo	27.8 ± 0.2	27.4 ± 0.2	**	–0.4 ± 0.1		27.9 ± 0.2		0.1 ± 0.2	
	LCPUFAs + LZ	27.4 ± 0.2	27.4 ± 0.2		0.0 ± 0.1	#	27.6 ± 0.2		0.2 ± 0.2	
SA (%)	Placebo	14.2 ± 0.2	14.4 ± 0.2		0.2 ± 0.1		14.3 ± 0.2		0.1 ± 0.1	
	LCPUFAs + LZ	14.5 ± 0.1	14.5 ± 0.2		0.0 ± 0.1		14.7 ± 0.2		0.2 ± 0.1	
OA (%)	Placebo	9.5 ± 0.2	9.7 ± 0.2		0.1 ± 0.2		9.7 ± 0.3		0.2 ± 0.2	
	LCPUFAs + LZ	9.3 ± 0.2	9.0 ± 0.2	**	–0.4 ± 0.2	#	9.1 ± 0.2		–0.2 ± 0.2	
LA (%)	Placebo	21.4 ± 0.4	21.5 ± 0.4		0.2 ± 0.4		21.6 ± 0.4		0.2 ± 0.4	
	LCPUFAs + LZ	21.0 ± 0.5	20.1 ± 0.5	**	–0.9 ± 0.3	#	20.3 ± 0.4		–0.6 ± 0.4	
ARA (%)	Placebo	9.0 ± 0.2	9.2 ± 0.3		0.2 ± 0.2		9.3 ± 0.3		0.3 ± 0.2	
	LCPUFAs + LZ	9.5 ± 0.2	10.2 ± 0.3	**	0.7 ± 0.2	#	10.2 ± 0.3	**	0.7 ± 0.2	
DHA (%)	Placebo	6.5 ± 0.3	6.5 ± 0.3		0.0 ± 0.2		6.5 ± 0.2		0.0 ± 0.2	
	LCPUFAs + LZ	6.6 ± 0.2	7.5 ± 0.2	**	1.0 ± 0.2	##	7.4 ± 0.2	**	0.8 ± 0.2	##
EPA (%)	Placebo	1.8 ± 0.2	1.9 ± 0.2		0.1 ± 0.2		1.9 ± 0.2		0.1 ± 0.2	
	LCPUFAs + LZ	1.9 ± 0.2	2.3 ± 0.2	*	0.4 ± 0.2		2.3 ± 0.1	*	0.3 ± 0.1	

Mean ± SE.

Placebo group (n = 36 at baseline, n = 35 at week 12, and n = 36 at week 24).

LCPUFAs + LZ group (n = 35 at baseline, n = 34 at week 12, and n = 34 at week 24).

There was no significant difference between groups at the baseline (unpaired Student's *t*-test).

*$p < 0.05$ and **$p < 0.01$ vs. baseline (paired *t*-test). #$p < 0.05$ and ##$p < 0.01$ vs. the placebo group (unpaired Student's *t*-test).

LCPUFAs, long-chain polyunsaturated fatty acids; LZ, lutein and zeaxanthin; PA, palmitic acid; SA, stearic acid; OA, oleic acid; LA, linoleic acid; ARA, arachidonic acid; DHA, docosahexaenoic acid; EPA, eicosapentaenoic acid.

Supplementary Table S5. Lutein and zeaxanthin concentration in serum by group during the intervention in Trial 1

	Group	Baseline	12 Weeks		Δ (12-Week Baseline)		24 Weeks		Δ (24-Week Baseline)	
Lutein (μg/mL)	Placebo	0.23 ± 0.02	0.24 ± 0.02		0.01 ± 0.02		0.24 ± 0.02		0.02 ± 0.01	
	LCPUFAs + LZ	0.27 ± 0.02	0.47 ± 0.03	**	0.20 ± 0.03	##	0.45 ± 0.03	**	0.19 ± 0.03	##
Zeaxanthin (μg/mL)	Placebo	0.12 ± 0.01	0.13 ± 0.01		0.01 ± 0.01		0.12 ± 0.01		0.00 ± 0.01	
	LCPUFAs + LZ	0.14 ± 0.01	0.20 ± 0.01	**	0.06 ± 0.01	#	0.19 ± 0.01	**	0.05 ± 0.01	#

Mean ± SE.

Placebo group (n = 36 at baseline, n = 35 at week 12, and n = 36 at week 24).

LCPUFAs + LZ group (n = 35 at baseline, n = 34 at week 12, and n = 34 at week 24).

There was no significant difference between groups at the baseline (unpaired Student's *t* -test).

**p < 0.01 vs. baseline (paired *t* -test). #p < 0.05 and ##p < 0.01 vs. the placebo group (unpaired Student's *t* -test).

LCPUFAs, long-chain polyunsaturated fatty acids; LZ, lutein and zeaxanthin.

Supplementary Table S6. Episodic memory tests by group at the baseline and at 24 Weeks in Trial 1

	Group	Baseline	24 Weeks		Δ	Δ adjusted
**Episodic memory**						
Composite memory	Placebo	94.9 ± 1.2	95.5 ± 1.3		0.5 ± 1.0	0.5 ± 1.0
	LCPUFAs + LZ	95.1 ± 1.0	97.4 ± 1.3		2.2 ± 1.1	2.3 ± 1.0
Verbal memory	Placebo	50.0 ± 0.7	50.9 ± 0.7		0.9 ± 0.8	0.7 ± 0.6
	LCPUFAs + LZ	50.9 ± 0.8	52.7 ± 0.8	**	1.8 ± 0.6	2.0 ± 0.6
Visual memory	Placebo	44.9 ± 0.8	44.6 ± 0.9		–0.3 ± 1.0	–0.1 ± 0.9
	LCPUFAs + LZ	44.2 ± 0.6	44.7 ± 0.9		0.4 ± 1.0	0.2 ± 0.9

Mean ± SE
Placebo (n = 36) and LCPUFAs + LZ (n = 34) groups.
For participants with missing values in episodic memory scores at week 24, baseline values were also removed.
There was no significant difference between groups at the baseline (unpaired Student's *t* -test).
$^{**}p < 0.01$ vs. baseline (paired *t* -test).
There was no significant difference in the changes (Δ) and changes adjusted by baseline (Δ adjusted) between groups (unpaired Student's *t* -test or analysis of covariance by baseline score).
LCPUFAs, long-chain polyunsaturated fatty acids; LZ, lutein and zeaxanthin.

Supplementary Table S7. Baseline characteristics of participants for subgroup analysis in Trial 1

	Placebo			LCPUFAs + LZ			*p*
Age (years)[a]	64.9	±	1.3	66.6	±	1.6	0.415
Sex (M/F)[b]	9/7			9/9			0.715
BMI (kg/m^2)[a]	22.2	±	0.4	23.5	±	0.8	0.173
Education (years)[a]	14.4	±	0.5	14.2	±	0.5	0.788
MoCA-J[a]	21.0	±	0.2	20.3	±	0.3	0.087
Composite memory[a]	94.4	±	1.8	91.9	±	1.4	0.288
Verbal memory[a]	49.2	±	1.2	48.5	±	1.2	0.673
Visual memory[a]	45.1	±	1.2	43.4	±	0.5	0.179
ARA in plasma PL (%)[a]	8.8	±	0.3	9.6	±	0.3	0.087
DHA in plasma PL (%)[a]	6.6	±	0.4	6.5	±	0.3	0.828
EPA in plasma PL (%)[a]	1.9	±	0.2	1.8	±	0.2	0.800

Mean ± SE

Placebo (n = 14 for episodic memory scores, n = 16 for all other data) and LCPUFAs + LZ (n = 18 for all data) groups.

For participants with missing values in episodic memory scores at week 12, baseline values were also removed.

There was no significant difference between groups in the baseline data for subgroup analysis (MoCA-J < 23) ([a]unpaired Student's *t* -test, [b]chi-square test).

PPS, per-protocol set; LCPUFAs, long-chain polyunsaturated fatty acids; LZ, lutein and zeaxanthin; BMI, body mass index;
MoCA-J, Montreal Cognitive Assessment Japanese version; ARA, arachidonic acid; DHA, docosahexaenoic acid;
EPA, eicosapentaenoic acid; PL, phospholipids

Supplementary Table S8. Dietary assessment by group for subgroup analysis during the intervention in Trial 1

	Group	Baseline				12 Weeks			Δ		
Energy (kcal/day)	Placebo	1587	±	117		1505	±	93	−64	±	83
	LCPUFAs + LZ	1702	±	130		1699	±	164	−3	±	85
Carbohydrate (g/day)	Placebo	204	±	17		194	±	15	−6	±	11
	LCPUFAs + LZ	198	±	16		206	±	17	8	±	9
Protein (g/day)	Placebo	57	±	5		58	±	4	1	±	4
	LCPUFAs + LZ	70	±	6		70	±	8	−1	±	5
Fat (g/day)	Placebo	52	±	5		48	±	4	−3	±	4
	LCPUFAs + LZ	62	±	5		60	±	8	−2	±	4
LA (mg/day)	Placebo	10572	±	935		9945	±	796	−360	±	860
	LCPUFAs + LZ	12213	±	969		11606	±	1418	−608	±	838
ALA (mg/day)	Placebo	1748	±	164		1602	±	130	−117	±	157
	LCPUFAs + LZ	1978	±	165		1867	±	242	−111	±	146
ARA (mg/day)	Placebo	145	±	14		143	±	12	−2	±	16
	LCPUFAs + LZ	193	±	16	#	194	±	23	1	±	13
DHA (mg/day)	Placebo	370	±	49		393	±	39	17	±	53
	LCPUFAs + LZ	596	±	72	#	561	±	75	−35	±	57
EPA (mg/day)	Placebo	207	±	30		224	±	24	11	±	33
	LCPUFAs + LZ	345	±	45	#	319	±	47	−25	±	38

Mean ± SE

Placebo (n = 16 at baseline, n = 15 at week 12) and LCPUFAs + LZ (n = 18 at baseline and week 12) groups.

#$p < 0.05$ vs. the placebo group (unpaired Student's *t*-test).

There was no significant difference between the baseline and 12-week data in each group (paired *t*-test).

There was no significant difference between groups in change (Δ) (unpaired Student's *t*-test).

LCPUFAs, long-chain polyunsaturated fatty acids; LZ, lutein and zeaxanthin; LA, linoleic acid; ALA, a-linolenic acid; ARA, arachidonic acid; DHA, docosahexaenoic acid; EPA, eicosapentaenoic acid.

Supplementary Table S9. Fatty acid composition of plasma phospholipid by group for subgroup analysis during the intervention in Trial 1

	Group	Baseline				12 Weeks				Δ			
PA (%)	Placebo	28.0	±	0.3		27.9	±	0.3		−0.2	±	0.2	
	LCPUFAs + LZ	27.2	±	0.3	#	27.0	±	0.3		−0.1	±	0.2	
SA (%)	Placebo	13.8	±	0.2		13.9	±	0.3		0.1	±	0.1	
	LCPUFAs + LZ	14.8	±	0.2	##	14.8	±	0.3		0.1	±	0.2	
OA (%)	Placebo	9.6	±	0.2		10.0	±	0.3		0.4	±	0.2	
	LCPUFAs + LZ	9.5	±	0.3		8.9	±	0.3	**	−0.6	±	0.2	##
LA (%)	Placebo	21.5	±	0.7		21.9	±	0.6		0.7	±	0.5	
	LCPUFAs + LZ	21.0	±	0.6		20.3	±	0.6		−0.7	±	0.4	
ARA (%)	Placebo	8.8	±	0.3		8.8	±	0.4		0.0	±	0.2	
	LCPUFAs + LZ	9.6	±	0.3		10.6	±	0.4	**	1.0	±	0.2	##
DHA (%)	Placebo	6.6	±	0.4		6.4	±	0.4		−0.3	±	0.2	
	LCPUFAs + LZ	6.5	±	0.3		7.4	±	0.3	**	0.9	±	0.2	##
EPA (%)	Placebo	1.9	±	0.2		1.7	±	0.2		−0.3	±	0.3	
	LCPUFAs + LZ	1.8	±	0.2		2.2	±	0.2		0.4	±	0.2	#

Mean ± SE.

Placebo (n = 16 at baseline, n = 15 at week 12) and LCPUFAs + LZ (n = 18 at baseline and week 12) groups.

**$p < 0.01$ vs. baseline (paired *t* -test). #$p < 0.05$ and ##$p < 0.01$ vs. the placebo group at the baseline or in change (Δ) (unpaired Student's *t* -test).

LCPUFAs, long-chain polyunsaturated fatty acids; LZ, lutein and zeaxanthin; PA, palmitic acid; SA, stearic acid; OA, oleic acid;
LA, linoleic acid; ARA, arachidonic acid; DHA, docosahexaenoic acid; EPA, eicosapentaenoic acid.

Supplementary Table S10. Lutein and zeaxanthin concentration in serum by group for subgroup analysis during the intervention in Trial 1

	Group	Baseline			12 Weeks				Δ			
Lutein (μg/mL)	Placebo	0.21	±	0.02	0.25	±	0.03		0.03	±	0.03	
	LCPUFAs + LZ	0.27	±	0.02	0.49	±	0.05	**	0.22	±	0.04	##
Zeaxanthin (μg/mL)	Placebo	0.11	±	0.01	0.13	±	0.01		0.02	±	0.02	
	LCPUFAs + LZ	0.14	±	0.01	0.20	±	0.02	**	0.06	±	0.02	

Mean ± SE.

Placebo (n = 16 at the baseline, n = 15 at week 12) and LCPUFAs + LZ (n = 18 at the baseline and week 12) groups.

There was no significant difference between groups at the baseline (unpaired Student's *t* -test).

**$p < 0.01$ vs. baseline (paired *t* -test). ##$p < 0.01$ vs. the placebo group (unpaired Student's *t* -test).

LCPUFAs, long-chain polyunsaturated fatty acids; LZ, lutein and zeaxanthin.

Supplementary Table S11. Episodic memory tests by group during the intervention for subgroup analysis in Trial 1

	Group	Baseline	12 Weeks		Δ		Δ adjusted	
**Episodic memory**								
Composite memory	Placebo	94.4 ± 1.8	89.6 ± 2.1	*	−4.8 ± 1.8		−4.5 ± 1.7	
	LCPUFAs + LZ	91.9 ± 1.4	95.4 ± 1.9	*	3.4 ± 1.4	##	3.2 ± 1.5	##
Verbal memory	Placebo	49.2 ± 1.2	47.1 ± 1.4		−2.1 ± 1.3		−2 ± 1.1	
	LCPUFAs + LZ	48.5 ± 1.2	50.8 ± 1.3	*	2.3 ± 0.8	##	2.2 ± 1.0	##
Visual memory	Placebo	45.1 ± 1.2	42.4 ± 1.3	*	−2.7 ± 1.2		−2.4 ± 1.1	
	LCPUFAs + LZ	43.4 ± 0.5	44.6 ± 1.0		1.2 ± 0.9	#	0.9 ± 1.0	#

Mean ± SE

Placebo (n = 14) and LCPUFAs + LZ (n = 18) groups.

For participants with missing values in episodic memory scores at week 12, baseline values were also removed.

There was no significant difference between groups at the baseline (unpaired Student's *t* -test).

**p* < 0.05 vs. baseline (paired *t* -test).

#*p* < 0.05 and ##*p* < 0.01 vs. placebo (unpaired Student's *t* -test or analysis of covariance by baseline score).

LCPUFAs, long-chain polyunsaturated fatty acids; LZ, lutein and zeaxanthin.

Supplementary Table S12. Dietary assessment by group during the intervention in Trial 2

	Group	Baseline			12 Weeks				Δ		
Energy (kcal/day)	Placebo	1710	±	56	1614	±	59	**	−96	±	34
	LCPUFAs + LZ	1695	±	58	1576	±	56	**	−119	±	35
Carbohydrate (g/day)	Placebo	224	±	8	211	±	8	*	−13	±	5
	LCPUFAs + LZ	220	±	9	201	±	8	**	−19	±	6
Protein (g/day)	Placebo	68	±	3	65	±	3		−3	±	2
	LCPUFAs + LZ	67	±	2	64	±	2	*	−3	±	1
Fat (g/day)	Placebo	55	±	2	51	±	2	**	−4	±	1
	LCPUFAs + LZ	55	±	2	52	±	2	*	−3	±	1
LA (mg/day)	Placebo	10397	±	441	9823	±	429	*	−575	±	272
	LCPUFAs + LZ	10488	±	399	10053	±	405		−436	±	273
ALA (mg/day)	Placebo	1647	±	78	1533	±	73	*	−114	±	47
	LCPUFAs + LZ	1648	±	70	1573	±	66		−76	±	50
ARA (mg/day)	Placebo	182	±	7	173	±	9		−8	±	6
	LCPUFAs + LZ	175	±	8	168	±	8		−7	±	5
DHA (mg/day)	Placebo	516	±	34	519	±	39		3	±	27
	LCPUFAs + LZ	481	±	27	466	±	25		−15	±	19
EPA (mg/day)	Placebo	296	±	22	300	±	26		4	±	18
	LCPUFAs + LZ	274	±	17	264	±	16		−10	±	12

Mean ± SE
Placebo (n = 87) and LCPUFAs + LZ (n = 90) groups.
For participants with missing values in BDHQ at the baseline or week 12, the other values were also removed.
There was no significant difference between groups at the baseline and in change (Δ) (unpaired Student's *t* -test).
**p* < 0.05 and ***p* < 0.01 vs. baseline (paired *t* -test).
LCPUFAs, long-chain polyunsaturated fatty acids; LZ, lutein and zeaxanthin; LA, linoleic acid; ALA, α-linolenic acid;
ARA, arachidonic acid; DHA, docosahexaenoic acid; EPA, eicosapentaenoic acid.

Supplementary Table S13. Baseline characteristics of the participants for subgroup analysis in Trial 2

	Placebo			LCPUFAs + LZ			p
Age (years)[a]	67.2	±	1.2	66.1	±	1.1	0.493
Sex (M/F)[b]	17/8			21/14			0.526
Residential area (Tokyo/Osaka)[b]	9/16			16/19			0.451
BMI (kg/m^2)[a]	22.9	±	0.4	23.0	±	0.6	0.958
Education (years)[a]	15.0	±	0.4	15.2	±	0.3	0.648
MoCA-J[a]	20.2	±	0.3	20.6	±	0.2	0.239
Composite memory[a]	93.5	±	2.0	91.3	±	1.5	0.380
Verbal memory[a]	49.0	±	1.2	47.6	±	0.9	0.371
Visual memory[a]	44.1	±	1.1	43.7	±	0.8	0.744
ARA in plasma PL (%)[a]	10.1	±	0.4	10.0	±	0.3	0.913
DHA in plasma PL (%)[a]	6.5	±	0.4	6.6	±	0.3	0.928
EPA in plasma PL (%)[a]	2.5	±	0.4	2.0	±	0.2	0.225

Mean ± SE
Placebo group (n = 24 for composite and verbal memory scores, n = 25 for all other data).
LCPUFAs + LZ group (n = 34 for composite and verbal memory score, n = 35 for all other data).
For participants with missing values in episodic memory scores at week 12, baseline values were also removed.

There was no significant difference between groups in the baseline data for subgroup analysis (MoCA-J < 23) in Trial 2 ([a]unpaired Student's *t* -test, [b]chi-square test).
PPS, per-protocol set; LCPUFAs, long-chain polyunsaturated fatty acids; LZ, lutein and zeaxanthin; BMI, body mass index;
MoCA-J, Montreal Cognitive Assessment Japanese version; ARA, arachidonic acid; DHA, docosahexaenoic acid; EPA, eicosapentaenoic acid; PL, phospholipids.

Supplementary Table S14. Dietary assessment by group for subgroup analysis during the intervention in Trial 2

	Group	Baseline				12 Weeks				Δ		
Energy (kcal/day)	Placebo	1796	±	129		1636	±	139	*	−160	±	68
	LCPUFAs + LZ	1727	±	89		1604	±	89	*	−123	±	57
Carbohydrate (g/day)	Placebo	235	±	19		214	±	20	*	−21	±	10
	LCPUFAs + LZ	232	±	14		208	±	12	*	−24	±	10
Protein (g/day)	Placebo	74	±	7		68	±	7		−6	±	3
	LCPUFAs + LZ	67	±	4		66	±	4		−1	±	2
Fat (g/day)	Placebo	56	±	4		49	±	5	*	−7	±	3
	LCPUFAs + LZ	54	±	4		52	±	4		−2	±	2
LA (mg/day)	Placebo	10680	±	896		10020	±	937		−660	±	538
	LCPUFAs + LZ	10454	±	627		9949	±	710		−504	±	452
ALA (mg/day)	Placebo	1688	±	152		1590	±	150		−98	±	89
	LCPUFAs + LZ	1615	±	107		1545	±	122		−71	±	82
ARA (mg/day)	Placebo	195	±	15		178	±	19		−17	±	13
	LCPUFAs + LZ	176	±	15		174	±	13		−2	±	10
DHA (mg/day)	Placebo	593	±	90		583	±	107		−10	±	66
	LCPUFAs + LZ	416	±	42		470	±	46		54	±	27
EPA (mg/day)	Placebo	349	±	61		347	±	70		−2	±	42
	LCPUFAs + LZ	227	±	27	#	264	±	29	*	37	±	18

Mean ± SE

Placebo (n = 24) and LCPUFAs + LZ (n = 34) groups.

For participants with missing values in BDHQ at the baseline or week 12, the other values were also removed.

#$p < 0.05$ vs. the placebo group at the baseline (unpaired Student's t-test). *$p < 0.05$ vs. baseline (paired t-test).

There was no significant difference between groups in change (Δ) (unpaired Student's t-test).

LCPUFAs, long-chain polyunsaturated fatty acids; LZ, lutein and zeaxanthin; LA, linoleic acid; ALA, α-linolenic acid;
ARA, arachidonic acid; DHA, docosahexaenoic acid; EPA, eicosapentaenoic acid.

Supplementary Table S15. Episodic memory tests by group during the intervention for subgroup analysis in Trial 2

	Group	Baseline	12 Weeks		Δ		Δ adjusted	
**Episodic memory**								
Composite memory	Placebo	93.5 ± 2.0	92.8 ± 1.8		−0.6 ± 1.4		0.0 ± 1.3	
	LCPUFAs + LZ	91.3 ± 1.5	95.4 ± 1.2	**	4.1 ± 1.4	#	3.7 ± 1.1	#
Verbal memory	Placebo	49.0 ± 1.2	48.9 ± 1.2		−0.1 ± 0.9		0.3 ± 0.9	
	LCPUFAs + LZ	47.6 ± 0.9	50.9 ± 0.8	**	3.2 ± 0.9	#	3.0 ± 0.7	#
Visual memory	Placebo	44.1 ± 1.1	43.4 ± 1.2		−0.7 ± 0.9		−0.6 ± 0.8	
	LCPUFAs + LZ	43.7 ± 0.8	44.5 ± 0.7		0.8 ± 0.7		0.8 ± 0.7	

Mean ± SE

Placebo group (n = 24 for composite and verbal memory scores, n = 25 for visual memory score).

LCPUFAs+LZ group (n = 34 for composite and verbal memory scores, n = 35 for visual memory score).

For participants with missing values in episodic memory scores at week 12, baseline values were also removed.

There was no significant difference between groups at the baseline (unpaired Student's *t* -test).

**p < 0.01 vs. baseline (paired *t* -test).

#p < 0.05 vs. placebo (unpaired Student's *t* -test or analysis of covariance by baseline score).

LCPUFAs, long-chain polyunsaturated fatty acids; LZ, lutein and zeaxanthin.
